# Minor defects of the luminal integrity in arterial introducer eSheaths after transcatheter aortic valve implantation

**DOI:** 10.1371/journal.pone.0176893

**Published:** 2017-05-08

**Authors:** Michael Buege, Till Koehler, Ralf Heiderhoff, Marc Papenheim, Si Wang, Heinrich Schleiting, Wolfgang H. Arnold, Jason R. Foerst, Melchior Seyfarth, Klaus Tiroch, Thomas Riedl, Marc Vorpahl

**Affiliations:** 1 Department of Cardiology, Zentrum für Forschung in der klinischen Medizin (ZFKM), HELIOS Klinikum Wuppertal, University Witten/Herdecke, Wuppertal, Germany; 2 School of Electrical, Information and Media Engineering, University of Wuppertal, Wuppertal, Germany; 3 Department of Biological and Material Sciences in Dentistry, Faculty of Health, School of Dentistry, Witten/Herdecke University, Witten, Germany; 4 Department of Cardiology, Carilion Roanoke Memorial Hospital, Virginia Tech Carilion School of Medicine, Roanoke, VA, United States of America; 5 Department of Cardiology, Heart Centre Bodensee, Konstanz and Witten/Herdecke University, Witten, Germany; Klinikum Region Hannover GmbH, GERMANY

## Abstract

**Background:**

Medical devices such as implant delivery systems are commonly used during minimally invasive procedures in the cardiovascular system. These devices often have lubricious polymer coatings to reduce friction between the device and blood vessels but coatings may separate and potentially cause serious injuries to patients.

**Methods:**

Lubricious coated eSheaths for transcatheter heart valve implantation were assessed for luminal integrity at the proximal, medial and distal part. We assessed the number, depths and area of luminal trails using environmental scanning electron microscope (ESEM), white light interferometry (WLI) and optical profilometry using area scale fractal complexity (asfc) as surface parameters. A total of 15 eSheaths were retrieved and analyzed after successful femoral transcatheter Sapien 3 implantation in patients (23 mm valve– 14F eSheath, 26 mm valve– 14F eSheath and 29 mm valve– 16F eSheath, n = 5 for each group). Unused eSheaths (14F and 16F) served as controls (n = 5 for each group).

**Results:**

ESEM revealed significantly greater number of trails after TAVR passage with the 23 mm, 26 mm and 29 mm valves compared to unused control 14F and 16F eSheaths (13.9 ± 3.1, 14.2 ± 2.3, 15.8 ± 1.7 vs. 0.08 ± 0.1 and 1.0 ± 0.5 [n]; p ≤ 0.0001 for all comparisons). Similarly, WLI showed minor, but significantly greater areas of luminal defects after 23 mm, 26 mm and 29 mm valve implantation vs. 14F and 16F unused controls (7.5 ± 0.9, 10.3 ± 1.1, 10.4 ± 1.4 vs. 4.1 ± 0.4 and 2.2 ± 0.4 [μm2], p = 0.0081). Likewise, the 3D-surface-measurement showed comparable results after implantation of the 23 mm, 26 mm and 29 mm valves vs. 14F and 16F unused control eSheaths (79.5 ± 6.3, 105.9 ± 5.3, 98.8 ± 4.8 vs. 5.1 ± 2.8 and 5.6 ± 0.5 [asfc] p = 0.0001).

**Conclusion:**

Measurable defects of the luminal layer occur during balloon expandable TAVR using 14F and 16F eSheaths though this is likely clinically insignificant. Further clinical investigations including a prospective assessment of minor peripheral embolization are needed to fully address the impact of this luminal defects.

## Introduction

Transfemoral transcatheter aortic valve replacement (TAVR) has proven superior to surgical aortic valve replacement for both high risk and intermediate risk patients [1, 2]. The current balloon expandable devices have improved their profile by using thinner alloys and offsetting the balloon from the stent during the crimping process to deliver through a low profile expandable sheath (eSheath). The current generation SAPIEN 3 (Edwards Lifescience, Irvine, CA) arterial eSheath consists of a flexible part which is placed into the femoral artery except for the outer sleeve at the very proximal part. The eSheath includes an ultra-low delivery profile with a dynamic expansion mechanism (DEM) and an outer hydrophilic coating. This sheath system expands transiently during the passage of the SAPIEN 3 transcatheter heart valve and refolds to a lower-profile diameter thereafter [3]. Hydrophilic and/or hydrophobic coated devices have been associated with serious adverse events after separation of the coatings of various intravascular devices by peeling, flaking, shedding, delaminating or sloughing [4, 5]. In 2014 Sanon S. et al. reported acute transcatheter aortic valve thrombosis after implantation of a first generation transcatheter heart valve with a fatal outcome, which was allegedly caused by hydrophilic polymer delamination from the introducer sheath [6].

The aim of this investigation was to study the luminal integrity of the eSheath after TAVR implantation with scanning electron microscopy (SEM), white light interferometry (WLI) and a standardized surface profilometry.

## Methods

### eSheath collection after TAVR

After successful transfemoral Sapien 3 TAVR a total of 15 eSheaths were flushed with buffered saline, purified with ultrasonic cleaning system and analyzed before disposal in 10/2014–10/2015. Five 14 French (F) eSheaths were prepared for analysis after implantation of 23mm Sapien 3 valves, five 14F eSheaths were prepared for analysis after implantation of 26 mm Sapien 3 valves and five 16F eSheaths were prepared for analysis after implantation of 29 mm Sapien 3 valves. Unused 14F eSheaths (n = 5) and 16 F eSheaths (n = 5) were analyzed as controls.

The study was approved by the local Ethics Committee (GZ: 105/2014) and all patients provided written informed consent.

### Preparation of eSheaths for analyzation of the luminal integrity

**Fig 1:**
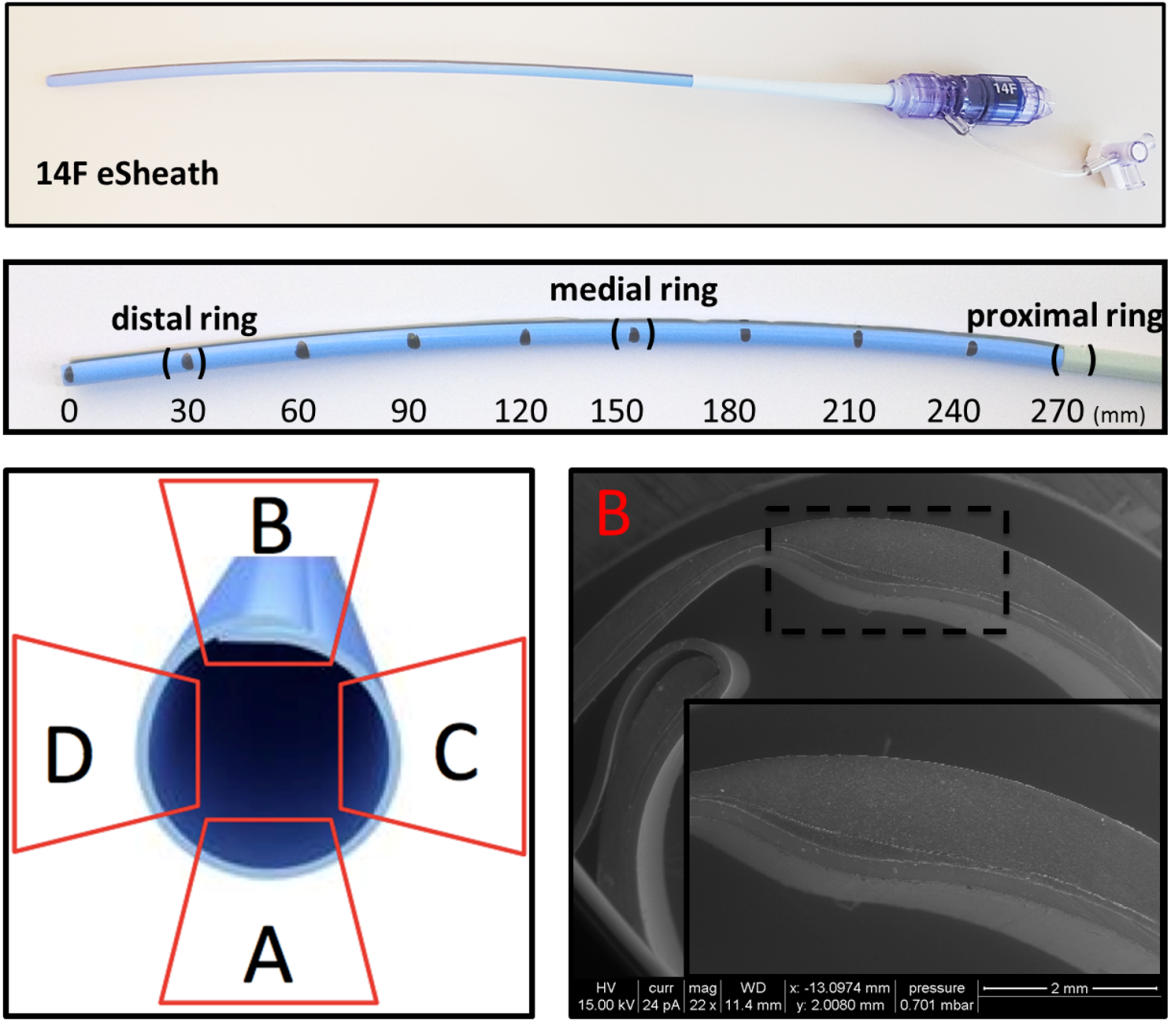
Preparation and assessment of the eSheath. The current generation eSheath for the Edwards SAPIEN 3 TAVR is a nominal 14F (23 and 26 mm valves) or 16F (29 mm valve) femoral sheath. The arterial sheath consists of a flexible part (360 mm) which is placed into the femoral artery (~270 mm) except for the outer sleeve at the very proximal part (~90 mm). A rigid proximal extracorporeal section includes the bleed-back prevention mechanism of the eSheath. eSheaths were cut by a medical scalpel in 5 mm rings at the proximal, medial and distal part of the eSheath. Four view fields (A, B, C, D) were assessed for the luminal integrity by ESEM, WLI and profilometry. ESEM cross section of the eSheath showed an inner, medial and outer polymer layer.

eSheaths were cut by a medical scalpel in 5 mm rings at the proximal, medial and distal part of the eSheath (Fig 1). Rings were flushed with 99% alcohol, air-dried and fixed on specimen holders for ESEM, WLI-analysis and profilometry. The cutting section was not included in the evaluation.

### Environmental scanning electron microscopy (ESEM)

Fixed 5 mm ring specimens were imaged with an environmental scanning electron microscope (Quanta 200, FEI) using a 15 kV acceleration voltage, 25 pA beam current, under 0.7 bar low vacuum condition, and an LFD detector (large field detector) to avoid charging. ESEM cross sections of the eSheath showed an inner, medial and outer polymer layer (Fig 1). Luminal integrity was defined by a scale from grade 0 (no trails), grade I (minor trails), grade II (moderate defect including the medial layer) to grade 3 (complete disruption of all layers).

### White light interferometry (WLI)

White light interferometry is a non-contact optical method for surface height measurement on 3-D structures. All 5 mm rings were cut into 4 parts which were fixed on sample holders. Those specimens were scanned and the coherence interferometry was analyzed by visible-wavelength light. This technology was utilized to determine the area of trails noted on ESEM.

### Profilometry

Surface roughness was assessed by an optical profilometer (MicroXAM 100 3D surface profilometer; InfiniteFocus, G3, Alicona Imaging GmbH, Graz, Austria) as described in detail previously [7, 8].

### Statistical analysis

Results are expressed as mean ± standard deviation. The significance of variability among the means of the experimental groups was determined by Wilcoxon-Mann-Whitney-Test. Each group was tested pairwise after Holm-Bonferroni method.

All statistical tests were performed by using the software JMP (Version 7. SAS Institute Inc., Cary, NC, 1989–2007). Differences among experimental groups were considered statistically significant at p < 0.05.

## Results

ESEM cross section of the eSheath demonstrated a three-layer composition with an inner layer, a thin intermediate layer and an outer layer (Fig 1). We focused the assessment of the luminal integrity on representative transverse sections of the flexible part of the eSheath (distal and medial ring) and the outer sleeve (proximal part).

### 1. Assessment of unused eSheaths

**Fig 2:**
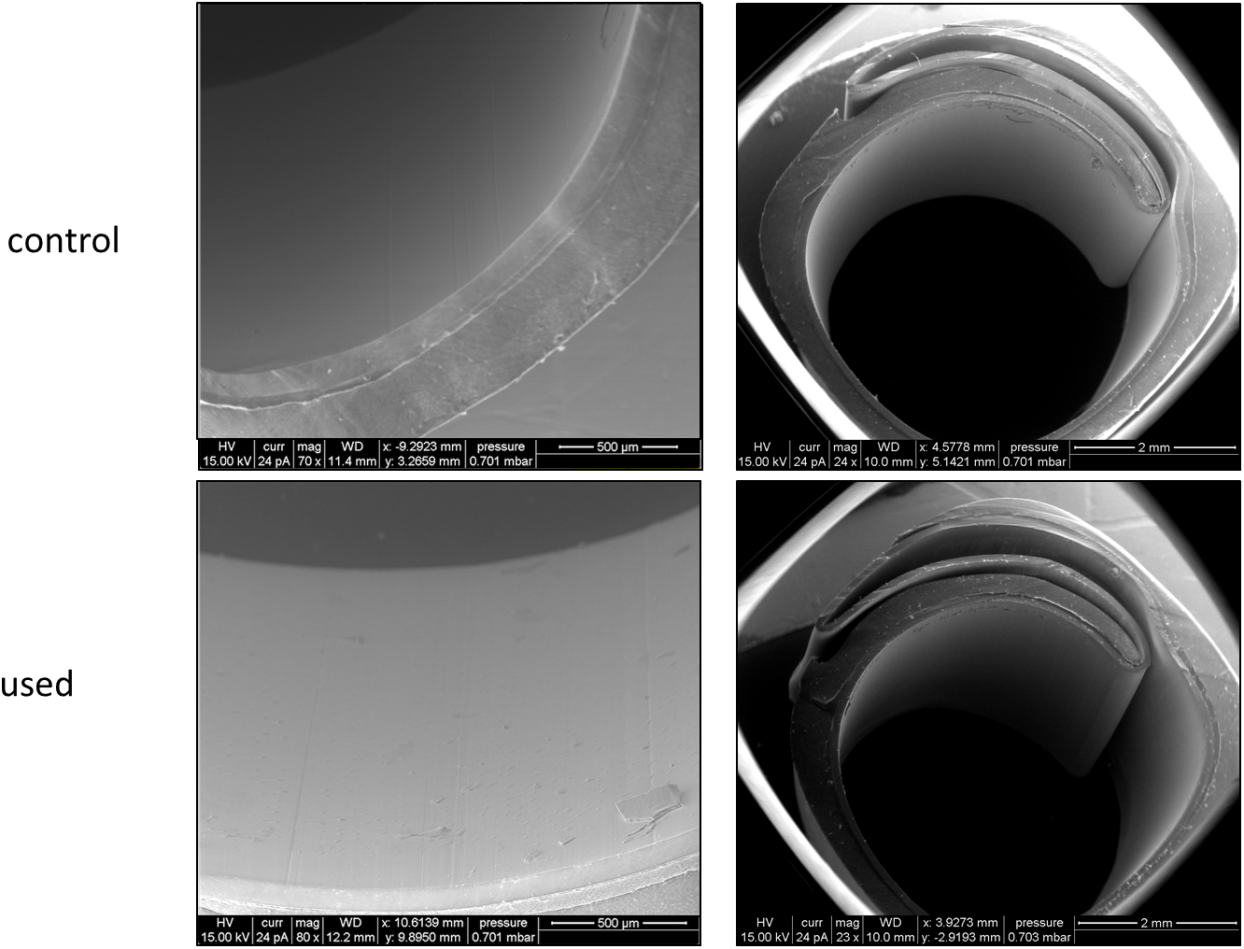
high power ESEM. Representative high and low power images of environmental scanning electron microscope of control and and used eSheaths.

**Fig 3:**
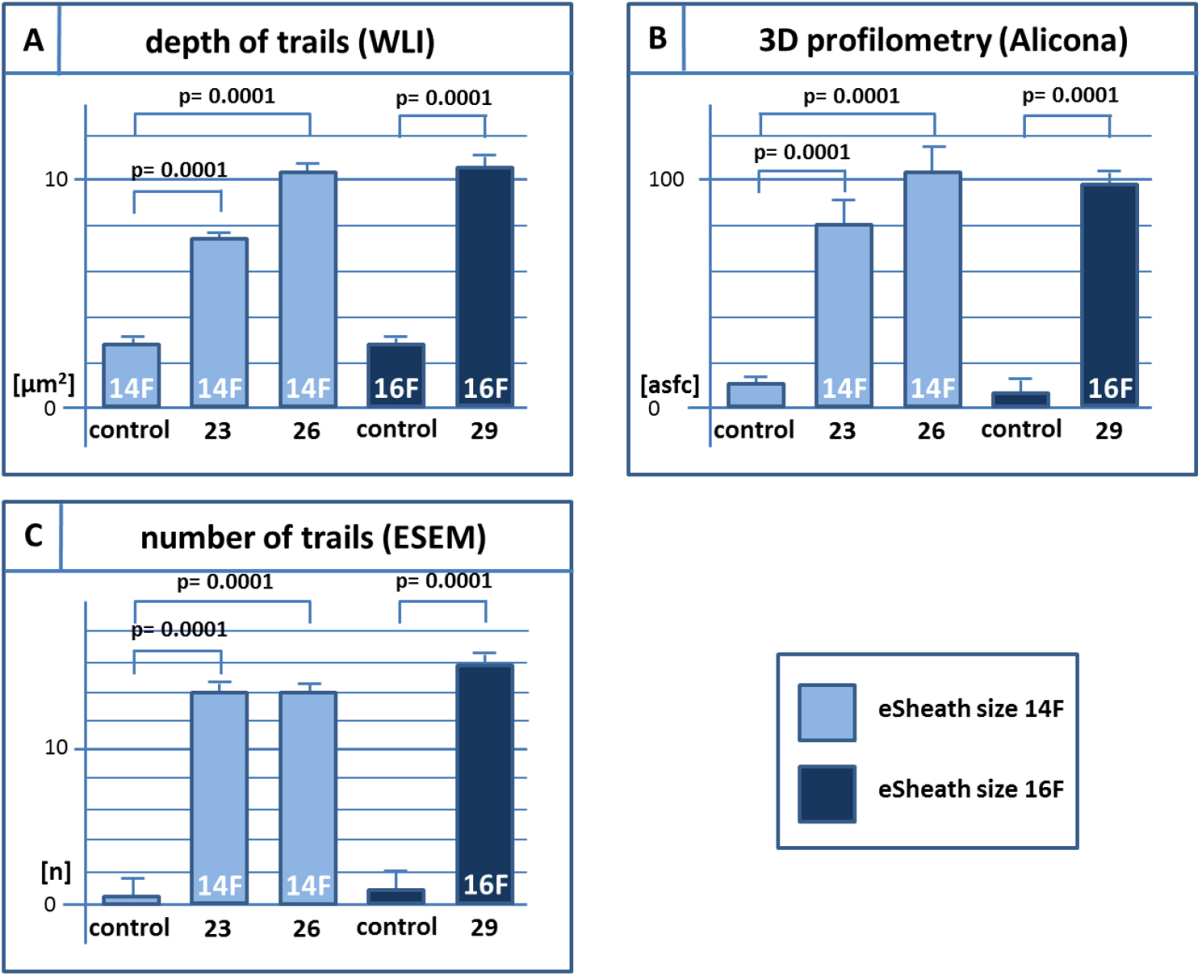
Results bar chart. Mean data of the number, depth and area of luminal trails using environmental scanning electron microscope (ESEM), white light interferometry (WLI) and optical profilometry using area scale fractal complexity (asfc) by a standardized 3D-surface-measurement system.

**Fig 4:**
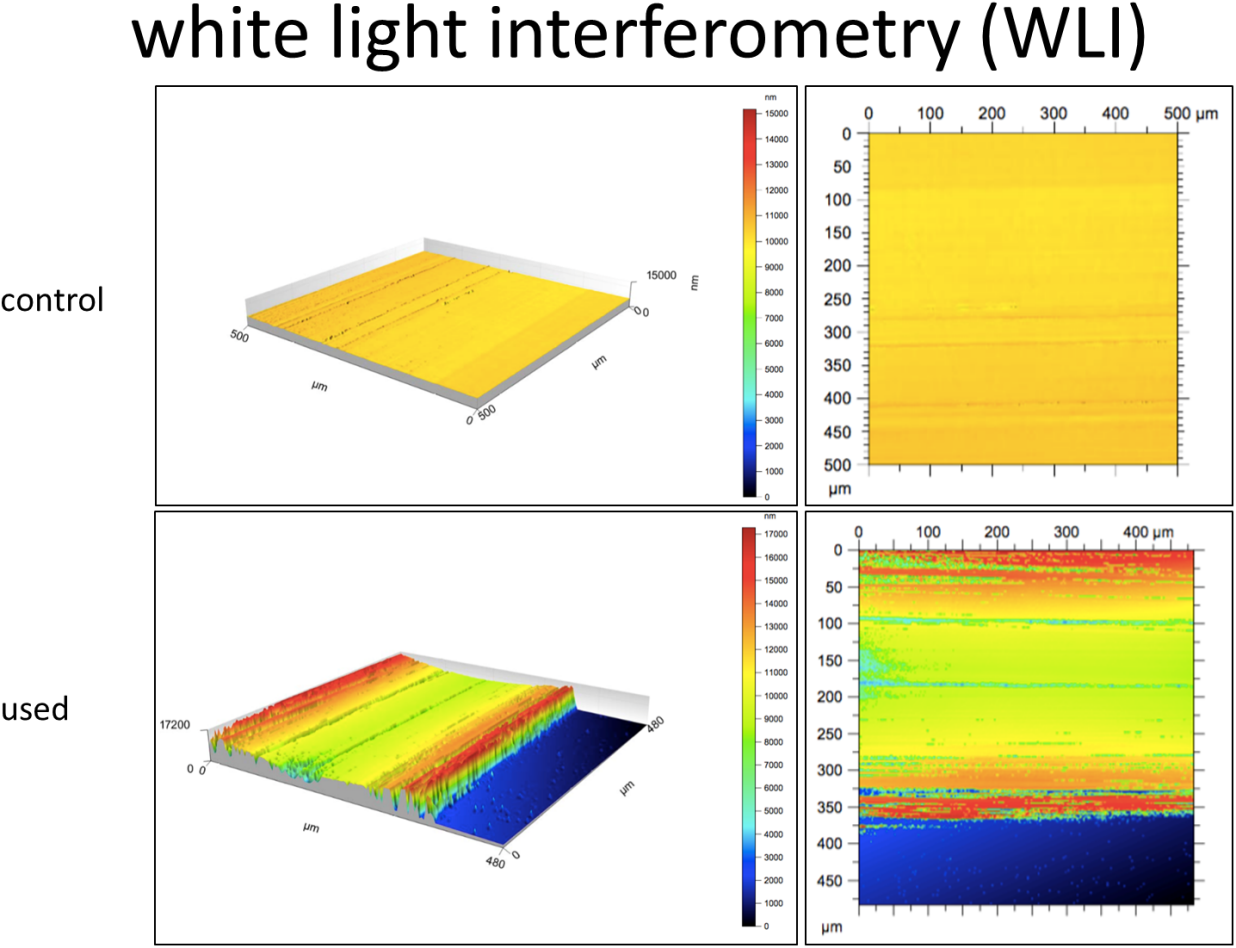
WLI. Representative high and low power images of environmental scanning electron microscope of control and and used eSheaths.

A total of five 14F eSheaths and five 16F eSheaths were analysed off-the-shelf. Representative cross sections of proximal, medial and distal rings were analyzed for the luminal integrity. The unused control eSheaths showed no trails (grade 0) in the vast majority of cases and scarce sections with minor trails (grade I). Count of trails was low with 0.8 ± 0.1 [n] for the 14F control eSheath and 1.0 ± 0.3 [n] for the 16F control sheath (Fig 2, Fig 3 and Table 1). WLI demonstrated similarly low areas of luminal defects in the 14F control eSheath (4.1 ± 0.4 μm2) and the 16F control eSheath (2.2 ± 0.4 μm2) (Fig 3, Fig 4 and Table 1). Both sheath sizes had similar profilometry roughness scores at 5.1 ±2.8 in the 14F control eSheath and 5.6 ± 0.5 [asfc] in the 16F control eSheath. Overall, both of sizes of control eSheaths were found to have luminal integrity with trivial defects only.

**Table 1:** Results data table of ESEM, WLI and Alicona in 14F and 16F eSheath.

size		ESEM (# trails)	WLI (depth of trails)	Alicona (asfc)
**14F**	23 mm valve	13.9 ±3.1	7.5 ±0.9 μm^2^	79.5 ±6.3
	26 mm valve	14.2 ±2.3	10.3 ±1.1 μm^2^	105.9 ±5.3
	unused control	0.08 ±0.1	4.1 ±0.4 μm^2^	5.1 ±2.8
16F	29 mm valve	15.8 ±1.7	10.4 ±1.4 μm^2^	98.8 ±4.8
	unused control	1.0 ±0.3	2.2 ±0.4 μm^2^	5.6 ±0.5

Table 1: Summary of the data for number, depth and area of luminal trails using environmental scanning electron microscope (ESEM), white light interferometry (WLI) and optical profilometry using area scale fractal complexity (asfc) by a standardized 3D-surface-measurement system.

### 2. Ex-vivo assessment of the eSheath luminal integrity post Sapien 3 femoral transcatheter valve implantation

eSheaths and TAVR were implanted with current good clinical practice at access sites with a minimal iliofemoral diameters > 8.0 mm with low to mild atherosclerotic burden, and low tortuosity. Collection of the eSheaths and Sapien 3 femoral transcatheter valve implantation procedures were uneventful.

We collected a total of five 14F eSheaths after 23 mm Sapien 3 implantation, five 14F eSheaths after 26mm Sapien 3 implantation and five 16F eSheaths after 29 mm Sapien 3 implantation.

**Fig 5:**
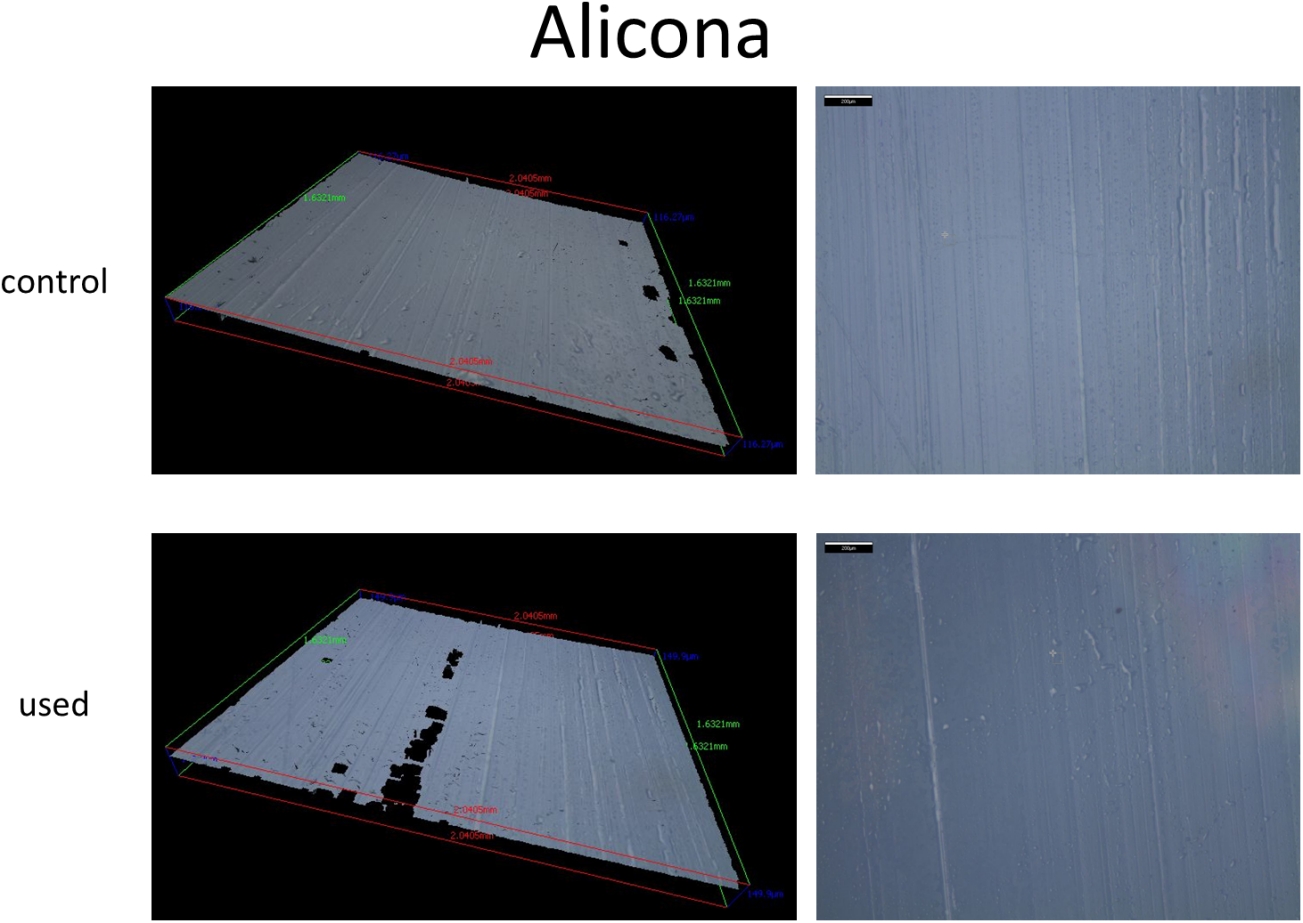
Profilometry. Representative high and low power images of optical profilometry using area scale fractal complexity of control and and used eSheaths.

ESEM revealed significantly greater numbers of trails after TAVR passage with the 23 mm, 26 mm and 29 mm valves (13.9 ± 3.1, 14.2 ± 2.3, 15.8 ± 1.7) compared to the control sheaths (0.08 ± 0.1 and 1.0 ± 0.3). The number of trails trend to increase with the valve size, but this was not statistically significant (Fig 2, Fig 5 and Table 1). The vast majority of sheaths had minor trails (grade I). We did not observe any sheaths with grade II trails (defect including the middle layer) or greater.

The results from the WLI were similar. Area of luminal defects were significantly greater after 23 mm, 26 mm and 29 mm valve implantation (7.5 ± 0.9, 10.3 ± 1.1, 10.4 ± 1.4 [μm2]) versus the control eSheaths (4.1 ± 0.4 and 2.2 ± 0.4 [μm2], p = 0.0081) and significantly greater after 26 and 29 mm valve implantation compared to 23 mm (p = 0.02) (Fig 3, Fig 4 and Table 1).

In addition, the 3D-surface-measurements demonstrated significantly greater defects of the luminal integrity after implantation of the 23 mm, 26 mm and 29 mm valves (79.5 ± 6.3, 105.9 ± 5.3, 98.8 ± 4.8) versus control eSheaths (5.1 ± 2.8 and 5.6 ± 0.5 [asfc], p = 0.0001). The surface roughness was significantly greater after 26 and 29 mm valve implantation compared to 23 mm valves (p = 0.0006) (Fig 3, Fig 5 and Table 1).

All eSheaths (100%) demonstrated some degree of alteration to the luminal integrity based on trails on ESEM and increased surface roughness after valve passage. Passage of 26 mm and 29 mm valves resulted in a significantly greater impact on luminal integrity compared to 23 mm valves.

## Discussion

To our knowledge this is the first luminal integrity analysis of commercially available expandable sheaths comparing unused control sheaths to comparable sheaths used in routine TAVR cases. This analysis demonstrates that the passage of the Sapien 3 valve through the recommended respective eSheaths results in significant alterations to the sheath luminal integrity that is directly proportional to the valve size.

Transcatheter aortic valve replacement (TAVR) is now approved for treatment of patients with severe, symptomatic aortic stenosis who are at least at intermediate risk for surgical aortic valve replacement. As the number of patients treated with TAVR increases, rare complications effect a far greater number of patients. Vascular complications during transfemoral TAVR occur around 10% of the time with modern delivery systems and can impact morbidity and mortality significantly [2, 9]. It is well understood that experience, femoral calcification, and the ratio of the sheath outer diameter to the minimal femoral artery diameter (sheath to femoral artery ratio—SFAR) have a significant impact on VARC2 major vascular complications [10–13]. On November 23, 2015 the FDA published a safety communication regarding “Lubricious Coating Separation from Intravascular Medical Devices”. The FDA wanted to make health care providers aware of the possibility that hydrophilic and/or hydrophobic coatings may separate (e.g., peel, flake, shed, delaminate, slough off) from medical devices and potentially cause serious injuries to patients. There has been at least one report of hydrophilic polymer delamination from the introducer sheath during TAVR leading to transcatheter aortic valve thrombosis after implantation of a first generation heart valve with a fatal outcome [6].

We sought to better understand the impact of valve delivery on the luminal side of these sheaths as well. Utilizing scanning electron microscopy (SEM), white light interferometry (WLI) and a standardized surface profilometry our study showed that the TAVR implantation leads to detectable defects of the luminal layer in currently used 14F and 16F eSheaths.

Scanning electron microscopy uses a focused beam of electrons to scan the surface of a sample with a resolution up to one nanometer. This technique provides high resolution images and is a widely accepted and useful tool for the safety assessment of intracoronary devices in ex vivo benchtop investigations [14] or preclinical testing [15]. Transection of the eSheath demonstrated a three-layer composition with an inner layer, a thin intermediate layer and an outer layer. Representative sections of the luminal surface showed significantly greater numbers of trails after TAVR passage with the 23 mm, 26 mm and 29 mm valves compared to the control sheaths. However, used and unused eSheaths were found to have only trivial defects of the luminal integrity.

White light interferometry is a non-contact surface topography instrument. The wave superposition principle is used to quantify the 3-D surface profile of a sample up to tens of nanometers. Our study demonstrated that the area of luminal defects were significantly greater after 23 mm, 26 mm and 29 mm valve implantation than in the control eSheaths. However, used and unused eSheaths were again found to have only trivial defects of the luminal integrity.

Profilometry by the Infinite Focus includes a 3D micro coordinate measurement machine and surface roughness measurement device in one system. Surface features are measured by a coaxial lightning and an optimized LED ring light which enables a resolution in nanometer scale. The determination of spatial loss provides a distinct insight e.g. into the effects on mineralized and demineralized tissue after abrasion of eroded dentin [16]. Our study demonstrated the 3D-surface-defects were significantly greater after implantation of the 23 mm, 26 mm and 29 mm valves compared to control eSheaths. However, used and unused eSheaths were again found to have only trivial defects of the luminal integrity.

**Fig 6:**
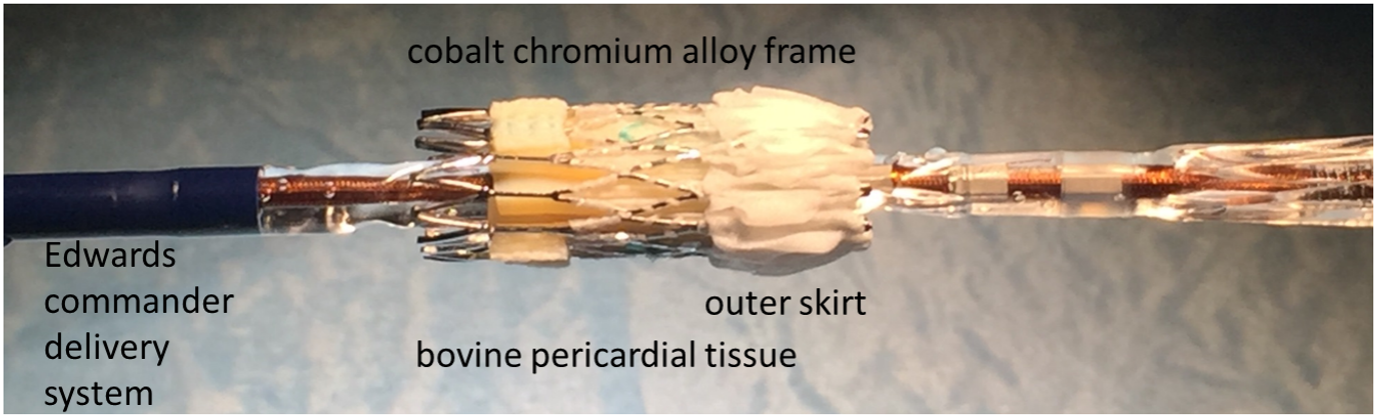
Current generation Sapien 3 valve. The current generation Sapien 3 valve includes a polymer outer skirt at the proximal edge of the valve. This may protect the cobalt chromium stent valve frame from the eSheath during valve passage.

The overall detected luminal defects involved rather minor trails and are unlikely to have a clinical impact. Of note we did not observe any significant defect of the medial or outer layer of the eSheath. This may be related to the modification of the new generation Sapien 3 valve including a polymer outer skirt (Fig 6). This may protect and separate the cobalt chromium stent valve frame from the eSheath during valve passage.

## Limitations

We performed an ex vivo study and did not assess any clinical/ in vivo event like the potential embolization in the blood stream. We did not assess the outer hydrophobic layer to determine the frequency of changes to the sheath after valve delivery because this layer may not be eligible to induce a proximal/ central embolization against the blood stream. Further clinical investigations including a prospective assessment of minor peripheral embolization are needed to fully address the impact of this luminal defects.

## Conclusion

The introduction of lower-profile sheaths has undoubtedly improved vascular complications rates as a major limitation of the TAVR procedure. However, further clinical investigations are needed to fully address the impact of this minor luminal defects.

## Supporting information

S1 File Summary of the local Ethics Committee approval. (DOC)

## Abbreviations

Asfc: area-scale-fractal-complexity
DEM: dynamic expansion mechanism
F: French
LPS: low-profile sheath
ESEM: environmental scanning electron microscope
TAVR: transcatheter aortic valve replacement
TAVI: transcatheter aortic valve implantation
WLI: white light interferometry
