# Supplementary material for: Minor defects of the luminal integrity in arterial introducer eSheaths after transcatheter aortic valve implantation
Source: PLoS One. 2017 May 8;12(5):e0176893. doi: 10.1371/journal.pone.0176893 (PMC5421765; doi:10.1371/journal.pone.0176893)
Supplement: S1 File — (DOC) [file pone.0176893.s001.doc]

Antragsteller:

Dr. med. Till Köhler

HELIOS Klinikum Wuppertal

Medizinische Klinik 3 - Kardiologie

Universität Witten/Herdecke

Arrenbergerstr. 20

42117 Wuppertal

Ethik-Kommission

der Universität Witten/Herdecke e.V.

z.Hd. Herrn RA Prof. Dr. med. P. Gaidzik

Alfred-Herrhausen-Straße 50

58448 Witten

**Antragsformular**

Der Antrag ist sorgfältig in deutscher Sprache auszufüllen. Er soll in kurzer, auch für die medizinischen Laien verständlicher Form das Vorhaben beschreiben und die Fragen durchgängig beantworten.

Für detaillierte Ausführungen muss im Antragsformular auf die Fundstellen in den Anlagen hingewiesen werden. Es muss sichergestellt sein, dass alle im Antragsformular gemachten Angaben auch in den Anlagen enthalten sind und den an der Prüfung beteiligten Ärzten und Wissenschaftlern zur Verfügung stehen. Das sind in der Regel:

- Prüfplan (datiert und vom Prüfleiter unterschrieben)
- Curriculum vitae des Leiters der klinischen Prüfung sowie des lokalen Prüfleiters, aus welchem ihre Erfahrungen mit klinischen Prüfungen hervorgehen
- Prüfbögen (CRF), gegebenenfalls als Entwurf
- Patienten-/Probandenaufklärung und -einwilligung
- Andere (z.B. Fachinformationen, Prüferinformationen, Monographien, Voten anderer Ethik-Kommissionen)
- Vergleiche auch die Geschäftsordnung der Kommission
- Bei Antrag auf Zweitvotum: Erstvotum der für den LKP zuständigen Ethikkommission

Die Unterlagen sind bei monozentrischen und für beantragte **Erstvoten** bei multizentrischen Studien in **siebenfacher**, bei einem beantragten **Zweitvotum** in **dreifacher** Ausfertigung - unter Beifügung des Erstvotums für den LKP - einzureichen.

***(Die Unterlagen sind gelocht, eingeheftet und unter Verzicht auf Büro- bzw. Heftklammern einzureichen!)***

Die Stellungnahme der Ethik-Kommission gilt nur für das Prüfvorhaben, wie es sich für die Kommission aus der ihr vorliegenden Fassung des Prüfplanes ergibt.

Die Prüfunterlagen werden vertraulich behandelt. Die Mitglieder der Ethik-Kommission sind kraft Gesetzes zur Verschwiegenheit verpflichtet.

A. Formales

**A1. Titel der klinischen Prüfung:**

► **Integritätstestung der Novaflexschleuse nach transfemoralem/ apikalen Aortenklappenersatz durch Rasterelektronenmikroskopie (Schleusen SEM)**

**A2. Lokal verantwortlicher Leiter der klinischen Prüfung:**

Name, Vorname, Titel: Köhler, Till, Dr. med.

| Jetzige Tätigkeit: | Assistenzarzt |
| --- | --- |

| Adresse: | Medizinische Klinik 3 - Kardiologie  HELIOS Klinikum Wuppertal  Arrenberger Str. 20  42117 Wuppertal |
| --- | --- |

Tel.-Nr.: 0202/896 5678

Email: till.koehler@helios-kliniken.de

**A3. Prüfstellen:**

Die Prüfung wird durchgeführt:

- ambulant

- stationär

Zahl der Prüfstellen bei Antragstellung, bitte Adresse(n) angeben: 1

Medizinische Klinik 3 - Kardiologie

HELIOS Klinikum Wuppertal

Arrenberger Str. 20

42117 Wuppertal

## Weitere Prüfer unter der lokal verantwortlichen Leitung:

| Name, Vorname, Titel: | Vorpahl, Marc, Dr. med. |
| --- | --- |
| Jetzige Tätigkeit: | Oberarzt |
| Adresse: | Medizinische Klinik 3 - Kardiologie  HELIOS Klinikum Wuppertal  Arrenberger Str. 20; 42117 Wuppertal |

| Name, Vorname, Titel: | Seyfarth, Melchior, Prof. Dr. med |
| --- | --- |
| Jetzige Tätigkeit: | Chefarzt |
| Adresse: | Medizinische Klinik 3 - Kardiologie  HELIOS Klinikum Wuppertal  Arrenberger Str. 20; 42117 Wuppertal |

| Name, Vorname, Titel: | Tiroch, Klaus, PD. Dr. med |
| --- | --- |
| Jetzige Tätigkeit: | Leitender Oberarzt |
| Adresse: | Medizinische Klinik 3 - Kardiologie  HELIOS Klinikum Wuppertal  Arrenberger Str. 20; 42117 Wuppertal |

| Name, Vorname, Titel: | Schleiting, Heinrich, Dr. med. |
| --- | --- |
| Jetzige Tätigkeit: | Oberarzt |
| Adresse: | Medizinische Klinik 3 - Kardiologie  HELIOS Klinikum Wuppertal  Arrenberger Str. 20; 42117 Wuppertal |

| Name, Vorname, Titel: |  |
| --- | --- |
| Jetzige Tätigkeit: |  |
| Adresse: |  |

**Nur bei Zweitvotierung:** Weitere verantwortliche Prüfleiter an anderen Prüfstellen: Anlage Nr.

**A4. Kostenträger (Sponsor, Rechnung für die Gebühr der Ethik-Kommission an):**

| Name: | Entfällt bei Studien außerhalb von AMG und MPG. |
| --- | --- |
| Anschrift: |  |

Gibt es einen Monitor?

nein

ja

**A5. Weitere Anträge**

Wurde nach Ihrem Wissen ein Antrag in der gleichen Sache bei einer anderen Ethik-

Kommission gestellt?

nein

ja

Falls ja, bitte Votum ggf. beifügen (Anlage Nr.     )

# B. Untersuchungsbeschreibung

**B1. Prüfplan**

- Liegt der vom lokalen Leiter der klinischen Prüfung unterschriebene Prüfplan bei?

nein

ja

Falls ja, Anlage Nr. 2

#### B2. Wissenschaftliche Kurzbeschreibung des Vorhabens mit Angabe der Prüfphase

- Prüfgerät / Prüfmethode / o.ä.:

Die katheter-gestützte Aortenklappenimplantation (TAVI) ist ein vielversprechendes neues Verfahren zur Behandlung von hochrisiko Patienten mit relevanter Aortenklappenverengung. Meistens wird die Prozedur durch die Leistenarterie (transfemoral) durchgeführt, kann aber auch über die Brustwand (transapikal) erfolgen. Zum Einbringen der Klappe in den Körper wird eine Schleuse verwendet die mit einer hypdrophilen Innenbeschichtung versehen ist. Die Schleusen sollen im Rahmen der Studie, nach dem Einsatz bei der Klappenintervention, mit dem Rasterelektronenmikroskop auf mögliche Beschädigungen oder Abrieb der Innenschicht, der durch das Einführen der Herzklappe verursacht wird, untersucht werden. Das Ausmaß der Beschädigung soll anhand einer Einteilung (Grad 1 bis 4) quantifiziert und eingeteilt werden. Des weiteren soll als Kontrollgruppe Schleusen von koronaren Stentimplantationen und nach Verschluss von Vorhofseptumdefekten und Vorhofohrverschluss, mit dem Rasterelektronenmikroskop untesucht werden.

- Neuentwicklung – bekannt – bekannt und zugelassen für die vorgesehene Indikation und Anwendungsform:

Die Edwards Sapien 3 Aortenklappenprothese sowie die Novaflex-Schleuse der Firma Edwards stellt weltweit das Standardverfahren beim transfemoralen und transapikalen Aortenklappenersatz dar. Es gibt jedoch bisher keine Veröffentlichung elektronenmirkoskopischer Untersuchungen nach dem Einsatz der oben genannten Schleusen.

- Studiendesign (offen – blind – doppelblind; vergleichend – randomisiert; monozentrisch – multizentrisch; prospektiv – retrospektiv; etc.):

Es handelt sich um eine offene, monozentrische, prospektive Untersuchung

- Biometrische Methode und spezielle statistische Auswertung (Gehen Sie bitte darauf ein, inwiefern die von Ihnen geplante Prüfung nach Art und Anlage eine wissenschaftlich und biometrisch begründete Aussage erlaubt und welche Hypothesen Sie prüfen wollen):

Hypothese: Das Einbringen der Herzklappe führt an der Innenschicht der Einführschleuse teilweise zu erheblichen Beschädigungen mit Abrieb von Scheusenmaterial und kann somit eine Gefahr für die Bildung von Blutgerinnseln (Thrombenbildung/Embolien) beim transfemoralen wie auch apikalen Zugang bei der katheter-gestützten Aortenklappenimplantation (TAVI) darstellen.

Randomisierung: keine

Patienten: Eingeschlossen werden alle Patienten mit schriftlicher Einverständnis und medizinisch-indizierter katheter-gestützten Edwards Sapien 3 Aortenklappenimplantation mit Novaflex-Schleuse über einen apikalen oder transaortalen Zugangsweg. Aktuell werden im Herzzentrum über diese Zugangswege etwa 120 Patienten pro Jahr behandelt.

ggf. siehe Prüfplan S. 8

#### B3. Vorgesehene Dauer der Prüfung

- Beginn der Studie: 01.09.2014

Ende der Studie: 30.10.2015

- Dauer pro Proband / Patient: 2 Monate

**B4. Probanden- / Patientenauswahl**

- Einschlusskriterien:

• Medizinisch indizierte transaortale oder apikale Implantation einer katheter-gestützten Aortenklappe

• Unterschriebene Studieneinverständniserklärung.

ggf. siehe Prüfplan S. 8

- Ausschlusskriterien:
(mit Angaben über Ausschlussbedingende Krankheiten und deren Stadien, verbotene Begleitmedikamente, Sperrfristen, Einwilligungsunfähigkeit, etc.)

• Alter <18 Jahren

• zeitgleiche Teilnahme an anderen Therapiestudien

ggf. siehe Prüfplan S. 8

- Stichprobenumfang: 20

**B5. Art der Prüfung**

diagnostisch

therapeutisch

epidemiologisch

Prävention

Sonstige:

**B6. Angewendete Bestimmungen**

Empfehlungen zur Planung und Durchführung von Anwendungsbeobachtungen vom 12.11.1998

Medizinproduktegesetz

Deklaration von Helsinki in der jeweils aktuellen, revidierten Fassung

Transfusionsgesetz

Strahlenschutzverordnung

Röntgenverordnung

Datenschutzgesetze

Sonstige:

**B7. Welche Vorprüfungen wurden am Menschen durchgeführt und mit welchem Ergebnis?**

- Bitte mit Quellenangabe und Beifügung wesentlicher Veröffentlichungen:

Coeytaux RR, Williams JW, Gray RN, Wang A (2010)

Percutaneous heart valve replacement for aortic stenosis: state of the evidence.

Ann Intern Med 153(5):314–324

Cribier A, Eltchaninoff H, Bash A et al (2002)

Percutaneous transcatheter implantation of an aortic valve prosthesis for calcific aortic stenosis: first human case description.

Circulation 106(24):3006–3008

Holmes DR Jr, Mack MJ (2011)

Transcatheter valve therapy: a professional society overview from the American College of Cardiology Foundation and the Society of Thoracic Surgeons.

Ann Thorac Surg 92(1):380–389

Vahanian A, Baumgartner H, Bax J et al (2007)

Task Force on the management of valvular hearth disease of the European Society of Cardiology, ESC Committee for Practice Guidelines. Guidelines on the management of valvular heart disease: the task force on the management of valvular heart disease of the European Society of Cardiology.

Eur Heart J 28(2):230–268

Vahanian A, Alfieri O, Al-Attar N et al (2008)

Transcatheter valve implantation for patients with aortic stenosis: a position statement from the European Association of Cardio-Thoracic Surgery (EACTS) and the European Society of Cardiology (ESC), in Collaboration with the European Association of Percutaneous Cardiovascular Interventions (EAPCI).

Eur Heart J 29(11):1463–1470

Sanon S, Maleszewski JJ, Rihal CS.

Hydrophilic polymer embolism induced acute transcatheter aortic valve thrombosis: A

novel complication. Catheter Cardiovasc Interv. 2014 Jun 1;83(7):1152-5. doi: 10.1002/ccd.25353. Epub 2014 Jan 29.

5

siehe auch Anlage Nr. 13

**B8. Bei Arzneimittelstudien:**

► Separates Antragsformular gemäß AMG und GCP-Verordnung in der derzeit gültigen Fassung verwenden!

**B9. Bei Medizinproduktestudien gemäß § 23 b MPG (**[**http://norm.bverwg.de/jur.php?mpg,23b**](http://norm.bverwg.de/jur.php?mpg,23b)**):**

- Sicherheitstechnischer Untersuchungsbericht und biologisch-toxikologische Prüfung bei bereits zugelassenen Medizinprodukten:

entfällt

- Nachweis des CE-Kennzeichens bzw. der Übereinstimmung mit der ISO 9000 durch einen „notified body“:

entfällt

***Für MPG-Studien außerhalb des § 23 b MPG und für In-vitro-Diagnostika:***

*Die Durchführung klinischer Prüfungen von Medizinprodukten und Leistungsbewertungsprüfungen von In-vitro-Diagnostika bedarf der Genehmigung der zuständigen Bundesoberbehörde und der zustimmenden Bewertung der zuständigen Ethik-Kommission.*

*Zur Einreichung von Anträgen auf Genehmigung der klinischen Prüfung gemäß § 22a MPG steht das zentrale Erfassungssystem des DIMDI zur Verfügung.*

[*http://www.dimdi.de/static/de/mpg/ismp/wegweiser/hinweise.htm*](http://www.dimdi.de/static/de/mpg/ismp/wegweiser/hinweise.htm)

**B10. Mögliche Komplikationen, Risiken und/oder Belastungen durch die geplante klinische Prüfung und ggf. vorgesehene Maßnahmen:**

- Kurzbeschreibung:

Die Studie vergleicht graduelle Unterschiede möglicher Beschädigungen und Abrieb an der Innenschicht von Einführschleusen beim kathetergestüzten Aortenklappenersatz.

ggf. siehe Prüfplan S. 8

##### B11. Nutzen-Risiko-Abwägung

- Gegenüberstellung von erhofftem Nutzen für den Patienten und/oder die Heilkunde und möglichen Risiken in Kurzbeschreibung:

Für den einzelnen Patienten ergeben sich aus der Teilnahme keine Risiken oder Nachtteile aber auch keine unmittelbaren Vorteile. Der wissenschaftliche Erkenntniswert dieser Studie besteht darin, durch die Identifizierung eines erheblichen Abriebes im Bereich der Schleuse einen Risikofaktor für unerwünschte Ereignisse wie z.B. eine Embolie durch Fremdmaterial zu identifizieren und damit ggf. zukünftig eine Verbesserung der TAVI Prozedur zu erreichen.

ggf. siehe Prüfplan S. 10

**B12. Zwischenauswertung / Abbruchkriterien**

- Ist eine Zwischenauswertung vorgesehen und welche Konsequenzen ergeben sich für den weiteren Versuchsablauf?

Bei dieser Studie ist aufgrund der kleinen Patientenzahl keine Zwischenauswertung vorgesehen.

- Welches sind Abbruchkriterien?

- für den Einzelfall

Widerruf der Einwilligung bzw. entsprechend der Ein- und Ausschlusskriterien.

- für die Prüfung insgesamt

entfällt

**B13. Aufklärung und Einwilligung**

Siehe dazu die bei der Ethik-Kommission erhältlichen "Hinweise zum erforderlichen Inhalt von Aufklärung und Einwilligung“

- Ist für die Aufklärung des Patienten die Übergabe eines Schriftstückes vorgesehen?

nein

ja

Falls nein, warum nicht:

Falls ja, Anlage Nr. 4

- Ist für die Einwilligung des Patienten ein Schriftstück vorgesehen?

nein

ja

Falls nein, warum nicht:

Falls ja, Anlage Nr. 4

**B14. Ist ein Informationsaustausch mit dem Hausarzt vorgesehen?**

nein

ja

Falls nein, warum nicht:

**B15. Erhält der Proband Aufwandsentschädigung?**

nein

ja

Falls ja, welche:

**B16. Angaben zum Versicherungsschutz des Versuchsteilnehmers gem. § 20 (3) MPG**

► Entfällt bei Studien außerhalb von AMG und MPG.

**B17. Besondere Bemerkungen**

*- Ich bin verpflichtet, die Ethik-Kommission über alle nachträglichen Änderungen des Prüfplanes und über alle schwerwiegenden oder unerwarteten unerwünschten Ereignisse, die während der Studie auftreten und die die Sicherheit der Studienteilnehmer oder die Durch­führung der Studie beeinträchtigen könnten, zu informieren. Ich werde dementsprechenden Mitteilungen meine eigene Bewertung der jeweiligen Ereignisse hinsichtlich Schwere und Kausalzusammenhang sowie eventuell eingeleitete oder geplante Maßnahmen oder Schlussfolgerungen hinzufügen.*

- Ich bin bereit, nach Abschluss des Vorhabens die veröffentlichten Ergebnisse der Ethik-Kommission zur Verfügung zu stellen.

Datum Unterschrift des lokalen Prüfleiters
